# Supplementary material for: CT features and histogram analysis of non-contrast images for differentiating malignant and benign mediastinal lymph nodes in Non-Small Cell Lung Cancer (NSCLC)
Source: PLoS One. 2025 Apr 17;20(4):e0321921. doi: 10.1371/journal.pone.0321921 (PMC12005500; doi:10.1371/journal.pone.0321921)
Supplement: S1 table — (DOCX) [file pone.0321921.s001.docx]

**Supplementary table 1:** The Cut-off Values and Diagnostic Performance of Histogram Parameters for Differentiating Benign and Malignant Lymph Nodes.

| Parameter | Cut-off | Sensitivity | Specificity | Youden's Index | Accuracy | LR+ |
| --- | --- | --- | --- | --- | --- | --- |
| Mean | 28.302 | 0.559 | 0.761 | 0.320 | 0.675 | 2.34 |
| Kurtosis | 40.866 | 0.529 | 0.804 | 0.334 | 0.688 | 2.71 |
| SD | 71.661 | 0.265 | 0.848 | 0.113 | 0.600 | 1.74 |
| Entrophy | 8.11 | 0.029 | 1.000 | 0.029 | 0.588 | 1.35 |
| MPP | 24.229 | 1.000 | 0.022 | 0.022 | 0.438 | 1.02 |
| Skewness | -19.325 | 1.000 | 0.000 | 0.000 | 0.425 | 1.00 |
