# Supplementary material for: CT features and histogram analysis of non-contrast images for differentiating malignant and benign mediastinal lymph nodes in Non-Small Cell Lung Cancer (NSCLC)
Source: PLoS One. 2025 Apr 17;20(4):e0321921. doi: 10.1371/journal.pone.0321921 (PMC12005500; doi:10.1371/journal.pone.0321921)
Supplement: S2 file — (PDF) [file pone.0321921.s003.pdf]

No.272/2022

Certificate of Ethical Approval

Name of Ethics Committee: Research Ethics Committee Panel 5
Faculty of Medicine, Chiang Mai University

Address of Ethics Committee: 110 Intavaroros Rd., Amphoe Mueang, Chiang Mai, Thailand 50200

Principal Investigator: Pakorn Prakaikietikul, M.D.
Department of Radiology, Faculty of Medicine, Chiang Mai University.

Protocol title: CT histogram for differentiating between malignant and benign mediastinal lymph nodes in the non-small lung cancers (NSCLC)

STUDY CODE: RAD-2565-09120 <u>Research ID</u>: 9120

Sponsor: -

Documents approved	Document reference
Research protocol	Version date.........4.....August 2022
Protocol amendment	-
Participant information sheet/ Informed consent form	-
Recruitment material	-
Case report form	Version date....4....August 2022
Patient's card and other documents given to research participants	-

Supplementary documents reviewed

Investigator's brochure -

Principal Investigator Curriculum vitae

- Pakorn Prakaikietikul, M.D. Version date.......4..August 2022

Co-Investigator Curriculum vitae

- Assist.Prof.Yutthaphan Wannasopha, M.D. Version date.......4...August 2022
- Assoc.Prof.Juntima Euathrongchit, M.D. Version date.......4...August 2022
- Assoc.Prof.Apichat Tantraworasin, M.D., Ph.D. Version date.......4.....August 2022

Certificate of Insurance -

The research has been approved by expedited review

No.272/2022

Date of Approval: ....4...... August 2022 **Expiration Date:** ....3......... April 2023

The Research Ethics Committee, Faculty of Medicine, Chiang Mai University is organized and operates according to Guideline for Good Clinical Practice (ICH GCP) and relevant international ethical guidelines, the applicable laws and regulations.

Signature : ......P. Kulapongs......

(Emeritus Professor Panja Kulapongs, M.D.)
Chairman of REC, Faculty of Medicine, CMU

POSTAPPROVAL REQUIREMENT:

- Investigator should make a request to renew approval within one month prior to the expiration date if the research is to be continued.
- In case there are any changes in the research protocol or informed consent form, the investigator must obtain REC approval prior to implementation unless (a) these changes are necessary for the safely of subjects, (b) minor changes such as logistical or administrative aspects of the trial (e.g., change of monitor(s), telephone number(s)).
- Any event or new information that adversely affects the safety of the subject or conduct of the trial must be reported to the REC promptly.
- Any protocol deviation/violation/noncompliance must be reported to the REC.
- Any serious adverse event must be reported to the REC promptly as stated in the Faculty of Medicine Notice.
