# Supplementary material for: CT features and histogram analysis of non-contrast images for differentiating malignant and benign mediastinal lymph nodes in Non-Small Cell Lung Cancer (NSCLC)
Source: PLoS One. 2025 Apr 17;20(4):e0321921. doi: 10.1371/journal.pone.0321921 (PMC12005500; doi:10.1371/journal.pone.0321921)
Supplement: Attachment — Submitted filename: Response to reviewers.docx [file pone.0321921.s005.docx]

Response to Reviewers

Dear Editors and Reviewers,

Thank you for allowing us to submit a revised version of our manuscript entitled “CT Features and Histogram Analysis for Differentiating Malignant and Benign Mediastinal Lymph Nodes in Non-Small Cell Lung Cancer (NSCLC)” for publication in “PLOS ONE”. We also thank the reviewers for their valuable comments that helped us improve our manuscript.

We have modified our manuscript incorporating the reviewers’ comments and we enclosed our response in a point-by-point manner.

We hope the revised manuscript will now be found suitable for publication in “PLOS ONE”. We look forward to hearing from you soon.

Reviewer #1: This manuscript explores an important area of radiology by attempting to use CT and histogram analysis to differentiate between malignant and benign lymph nodes in NSCLC. Below are specific comments and suggestions for improvement:

1. Size Measurement: Please clarify whether the lymph node size parameter refers to the long-axis or short-axis measurement.

Answer: Thank you for your insightful comment. In our study, the short-axis measurement was used to represent lymph node size. This clarification has been added to the manuscript in the Materials and Methods section on page 5, line 93-94.

2. In the Results section, the reporting of size statistics is inconsistent, with benign nodes described using median and malignant nodes using mean. Please standardize these metrics.

Answer: Thank you for your attention to this detail, and I apologize for the mistake. To ensure consistency, we have used the median to represent the average size of both benign and malignant nodes, as the data for both populations do not follow a normal distribution. This adjustment has been made in the Results section to maintain uniformity and accurately reflect the statistical characteristics of the data.

3. How was necrosis diagnosed in this study? As non-contrast CT is inherently limited in identifying necrosis, this methodology requires clarification and justification.

Answer: Thank you for your insightful comment regarding the diagnosis of necrosis. Necrotic regions within lymph nodes manifest as areas of low attenuation on non-contrast CT scans, appearing less dense than surrounding tissues. This characteristic reduction in density is suggestive of necrosis. In our study, we identified necrosis by observing these focal low-attenuation areas within lymph nodes on non-contrast CT images. This clarification has been added to the manuscript in the Materials and Methods section on page 5, line 95-96, along with the relevant code references.

4. Was the evaluation performed by a single radiologist? To improve reliability, it is recommended that assessments be conducted by at least two radiologists, with an analysis of inter-observer agreement.

Answer: Thank you for your valuable feedback. In our study, the preoperative CT images of mediastinal lymph nodes were independently and blindly reviewed by two board-certified diagnostic radiologists. The evaluation focused on the following characteristics: size, shape, margins, and internal composition.

The inter-observer agreement has been analyzed and added to the manuscript in the Results section on page 8, line 143-147, and is presented in Table 2.

5. Please provide details on the statistical methods used for the multivariate analysis in the Materials and Methods section.

6. The multivariate analysis includes only histogram parameters. Would it not be more robust to include all parameters with significant differences in univariate analysis?

Answer question number 5 and 6: Thank you for your valuable comments. We acknowledge the inaccuracies in our initial description of the statistical analysis. In diagnostic studies, multivariable analysis is typically employed to evaluate the collective predictive value of all variables or to identify key factors for inclusion in clinical prediction models.

However, in our study, the purpose of the analysis was not to develop a predictive model or assess all significant variables from the univariate analysis. Instead, we conducted Receiver-operating characteristic (ROC) curve analysis to calculate the area under the curve (AUC) for CT texture histogram parameters, focusing on their ability to distinguish malignant from benign mediastinal lymph nodes. This clarification has been incorporated into the Results section on page 9, lines 170-173 and is presented in Fig 3.

7. For the histogram analysis parameters, diagnostic cut-off values, sensitivity, specificity, and overall diagnostic accuracy should be provided to enhance the clinical applicability of your findings.

Answer: Thank you for your insightful feedback. Upon further analysis, we have revised the cut-off values for nodal size and volume. The cut-off for nodal size has been adjusted from 10.5 mm to 13.0 mm, and for nodal volume from 1.47 ml to 1.63 ml. These new cut-off values were selected because they are more strongly supported by Youden's Index and the positive likelihood ratio (LR+), making them the preferred indicators for malignancy. These have been added to the manuscript in the Results section on page 10, line 181-187, and is presented in Table 4.

For the remaining histogram parameters, our analysis revealed consistently low sensitivity, specificity, accuracy, Youden’s Index, and LR+, indicating limited utility in predicting malignancy. As a result, we have chosen to highlight only the cut-off values for size and volume in the main manuscript, while the full set of histogram parameter cut-offs is presented in the supplementary table.

We believe this approach enhances the clarity and clinical relevance of the manuscript. Please let us know if you would like further clarification or if additional revisions are necessary.
